# Models for Heart Failure Admissions and Admission Rates, 2016 through 2018

**DOI:** 10.3390/healthcare9010022

**Published:** 2020-12-27

**Authors:** Clemens Scott Kruse, Bradley M. Beauvais, Matthew S. Brooks, Michael Mileski, Lawrence V. Fulton

**Affiliations:** Department of Health Administration, Texas State University, San Marcos, TX 78666, USA; scottkruse@txstate.edu (C.S.K.); bmb230@txstate.edu (B.M.B.); mbrooks@txstate.edu (M.S.B.); mileski@txstate.edu (M.M.)

**Keywords:** heart failure, geographical, obesity, cost analysis

## Abstract

Background: Approximately 6.5 to 6.9 million individuals in the United States have heart failure, and the disease costs approximately $43.6 billion in 2020. This research provides geographical incidence and cost models of this disease in the U.S. and explanatory models to account for hospitals’ number of heart failure DRGs using technical, workload, financial, geographical, and time-related variables. Methods: The number of diagnoses is forecast using regression (constrained and unconstrained) and ensemble (random forests, extra trees regressor, gradient boosting, and bagging) techniques at the hospital unit of analysis. Descriptive maps of heart failure diagnostic-related groups (DRGs) depict areas of high incidence. State- and county-level spatial and non-spatial regression models of heart failure admission rates are performed. Expenditure forecasts are estimated. Results: The incidence of heart failure has increased over time with the highest intensities in the East and center of the country; however, several Northern states have seen large increases since 2016. The best predictive model for the number of diagnoses (hospital unit of analysis) was an extremely randomized tree ensemble (predictive R^2^ = 0.86). The important variables in this model included workload metrics and hospital type. State-level spatial lag models using first-order Queen criteria were best at estimating heart failure admission rates (R^2^ = 0.816). At the county level, OLS was preferred over any GIS model based on Moran’s I and resultant R^2^; however, none of the traditional models performed well (R^2^ = 0.169 for the OLS). Gradient-boosted tree models predicted 36% of the total sum of squares; the most important factors were facility workload, mean cash on hand of the hospitals in the county, and mean equity of those hospitals. Online interactive maps at the state and county levels are provided. Conclusions. Heart failure and associated expenditures are increasing. Costs of DRGs in the study increased $61 billion from 2016 through 2018. The increase in the more expensive DRG 291 outpaced others with an associated increase of $92 billion. With the increase in demand and steady-state supply of cardiologists, the costs are likely to balloon over the next decade. Models such as the ones presented here are needed to inform healthcare leaders.

## 1. Introduction

### 1.1. Demand for Treatment

Heart failure (HF) is increasing in prevalence and affects at least 26 million adults globally [1] and 6.5 million [2] to 6.9 million adults in the United States [3]. In men and women aged 64 to 75, five-year mortality mean estimates range from 40 to 71% [4]. One out of 8 deaths in 2017 was attributed at least in part to HF, and the annualized cost was estimated to be $30.7 billion in 2012 [2], rising to $43.6 billion in 2020 [3]. HF is a subset of coronary heart disease (CHD), which is the leading cause of death in most developed countries [5].

Age and male gender are known risk factors for HF [6,7] along with hypertension, left ventricular hypertrophy, myocardial infarction, diabetes mellitus, valve disease, and overweight or obese status [8]. The increased risk for males may be partially explained by greater incidence and prevalence of coronary heart disease (CHD) [9,10]. Less consistently associated risks for HF include alcohol consumption, cigarette smoking, dyslipedemia, renal insufficiency, sleep-disordered breathing, low physical activity, low socioeconomic status, coffee consumption, dietary sodium intake, increased heart rate, impaired pulmonary function, and mental stress/depression [8]. 

Chronic HF consumed an estimated 1–2% of total healthcare resources in industrialized nations in 2004 [11], yet HF and CHD were not common causes of death at the turn of the 20th century [12]. Between 1974 and 2004, hospitalizations for HF more than tripled in the United States, rising from 1.28 million to 3.86 million [13]. The increase is partially explainable due to the aging of the population [14]. The economic impact of heart failure was estimated to be $108 billion per annum in a study of 197 countries [15].

### 1.2. Supply and Payment of Cardiologists

Despite the national average of 383 people per physician in the United States, the number of people per cardiologist is 14,572 [16]. There is certainly an element of artificiality in those numbers because while all people in the U.S. seek some medical care, a much smaller number need specialty care from a cardiologist. However, the message is the same: cardiology is highly specialized and a highly sought area of care. 

While the general trend is up for cardiovascular disease (CVD), the growth of those entering cardiology is relatively flat. It is estimated that 40.5% of the U.S. population will have some form of CVD by 2030. This equates to a 3.1% incidence rate and $818 billion in cost of care [17]. A 2018 study of HF incidence from 1990 to 2009 revealed that HF with reduced ejection fraction (HFrEF) was down, while HF with preserved ejection fraction was up (HFpEF) [18]; see Appendix A for a list of abbreviations. More recent studies are not readily available.

### 1.3. Relevant Methods

Explanatory models for healthcare costs have included linear and penalized linear models such as a lasso regression [19] with reasonable success. Other machine learning techniques such as random forests have also been used to predict and explain CHD events and risk factors successfully [20]. Random forests are an ensemble of tree models used for either regression or classification [21]. This study uses these models for explanatory investigation of CHD in this study as well, as they have proven successful in previous studies of this nature. 

Geospatial analyses are becoming increasingly important and relevant in the area of public health [22]. With visualization and analytical capabilities, geospatial analyses in public health are now ubiquitous [23,24,25,26,27,28,29,30,31]. A review of geospatial methods used for public health as well as the historical development is available from Saran, Singh, Kumar, and Chauhan [22]. 

Specific research in public health has leveraged geospatial analysis to look at several aspects of heart disease such as emergency transport and interhospital transfer of myocardial infarction [32] as well as individual and contextual correlates of cardiovascular disease [33]. Spatial analysis in the area of public health is conducted at the worldwide, country, and regional levels of analysis [34]. Most often, choropleth maps are used to present one or two data attributes, although dot maps, graduated symbol maps, and isarithmic maps are also commonplace [34]. Spatial regression techniques such as simultaneous autoregressive (SAR) models are often used to document health risks [35], and spatial clustering has been used for leprosy in Brazil [36], measle vaccination in sub-Saharan Africa [37], as well as food and physical activity in the United States [38]. Spatial analysis has been used to identify congenital heart disease in youth aged 4–18 in China as well [39]. Geospatial analysis has also been used for describing birthing incidence [40], the opioid epidemic [41], evaluating back surgery growth over time [42], and in many other health-related studies. To date, however, researchers have not conducted geospatial analyses of HF with predictive modeling to provide epidemiological and administrative descriptive and inferential insight as well as economic implications for supply and demand. This research does just that over a three-year window (2016 through 2018). 

### 1.4. Research Question and Significance

This research seeks to understand the geospatial incidence of CHD by state and county and to build predictive models that forecast hospitals’ number of HF DRGs using technical, workload, financial, and geospatial variables. Analysis and forecasting of the raw numbers of HF DRGs provides for financial and demand estimation based on empirical inflationary pressures and existing/forecast disease rate increases. Understanding patterns is important to both policy makers, epidemiologists, and health administrators alike for cost control and planning efforts. Finally, the demand and supply analysis highlight potential shortfalls that may require redress. 

## 2. Materials and Methods

### 2.1. Data

Data from this study come from the Definitive Healthcare dataset [43]. Diagnostic-related groups (DRGs) associated with HF (DRGs 291, 292, and 293) were selected for inclusion. The Definitive Healthcare datasets contain the Centers for Medicare and Medicaid Services (CMS) Standard Analytical Files (SAF) [43]. State and county-level population data for rate calculations were from the Census Bureau [44,45]. For years 2016 through 2018, there were 13.66, 13.52, and 13.35 thousand hospital observations in the study, respectively. These hospital observations were associated (respectively) with 20.08, 22.74, and 23.46 million DRGs. For the geospatial analyses only, the DRG counts were aggregated by county and state for different analyses under the assumption that there might be a geographical relationship. These counts were then converted to rates based on the population of the geographic unit, as rates per population base provide a comparison basis across geographical units.

### 2.2. Variables

The primary variable of interest is admissions for “heart failure” diagnoses as defined by diagnostic-related groups 291, 292, and 293 [46]. Diagnostic-related group 291 encompasses “Heart Failure and Shock with Major Complication of Comorbidity (MCC)”; DRG 292 relates to “Heart Failure and Shock with Complication or Comorbidity (CC); DRG 293 pertains to “Heart Failure and Shock without Complication or Comorbidity (CC)/Major Complication or Comorbidity”. The dependent variable is measured at the hospital level and aggregated by county for l mapping. Inpatient claims for HF provide a measure of the met demand for services and is suggestive of which areas may need additional funding and resources from health policy decision makers. 

Variable groups evaluated in the explanatory models included four categories: financial variables, workload variables, technical variables, and geospatial temporal variables. All variables are measured at the hospital level by year. Table 1 provides the definitions of the independent variables.

### 2.3. Models for Number of Heart Failure Admissions

#### 2.3.1. Train and Test Sets

For the non-spatial model exploration, data were divided randomly using a pseudo-random seed for replication and consistency in model comparison into 80% training and 20% test set of sizes 32,206 and 8051, respectively. Models were built on the training set and evaluated on the test set. The splitting of the data occurred prior to any imputation or transformations, so that no information would be leaked from one set to the other. The primary model selection metric for non-spatial models was R^2^, the proportion of the sum of squares accounted for by variables in the model. 

#### 2.3.2. Imputation, Transformation, and Scaling

Little data were missing (2%). Observations with 33% or more missing data were deleted. Imputation was conducted separately for the total data, the training data, and the test data. Six workload-related variables (discharges, emergency room visits, surgeries, staffed beds, affiliated physicians, and employees) were highly collinear and replaced with a single principal component that accounted for 84.8% and 84.9% of the variability in the total and training datasets, respectively. The workload variable for the test set was built using the linear combination estimated on the training set to avoid information leakage between the two data sets. Data were then scaled between zero and unity, as some models are not scale invariant. 

#### 2.3.3. Explanatory Analysis for the Number of Heart Failure Diagnoses

Linear regression, lasso regression, robust regression, elastic net regression, random forests, extra trees random forests, extreme gradient-boosted random forests, and bagging regressors estimate the DRG HF admissions. To investigate the bias–variance trade-off [47], we built multiple models on an 80% training and evaluated on a 20% test set. The models are exploratory to see which features (workload, financial, technical, and geospatial) might be explanatory. 

Lasso regression is a constrained regression that penalizes overfitting using an L1-norm penalty function (absolute value), while ridge regression is similar to lasso regression but penalizes using the L2-norm (squared) [47]. Elastic net combines both lasso and ridge penalty functions [48]. While coefficients are easily interpreted in regression-type models, the data typically require scaling and transformations. Unlike tree ensemble models (forests), regression models are unable to find polytomous splits of variables automatically and are not scale invariant. To address the concerns of collinearity, principal component analysis is performed. 

#### 2.3.4. Tree Models

**Figure 1:**
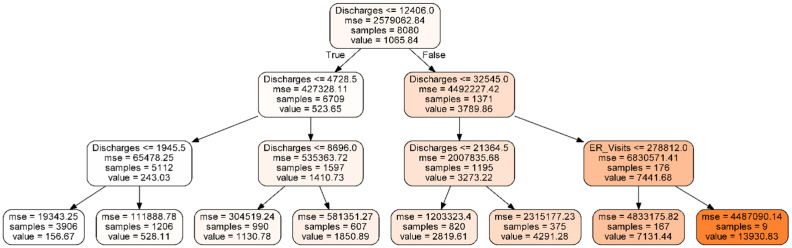
An example of a tree model to classify opioid admissions.

Random forests are an ensemble of de-correlated tree models. Figure 1 is an example of a tree with three branches that includes a random subset of candidate features (variables). The tree splits observations by the number of hospital discharges less than or equal to versus greater than or 12,406 initially to obtain the maximum separation (RMSE). In a random forest, every tree produces a separate forecast. All trees produced are than averaged to produce the estimate. Trees are “pruned” to prevent overfitting [47]. 

Extremely randomized regression trees (extra trees) add randomness by generating random split locations for features and using the best threshold as the splitting rule [49]. These models typically result in less variance but higher bias. 

Gradient boosting is an ensemble of weighted trees composed by iteratively assigning weights to trees that reduce prediction error [50]. These models use non-linear optimization to optimize a cost function based on the (pseudo)-residuals of a given function. Unlike random forests, gradient-boosted random forests do not produce uncorrelated trees. Instead, the residuals of each tree are re-fitted with the possible independent variables in other tree models. A more complete discussion of gradient boosting is provided in *The Elements of Statistical Learning* [47].

A bagging tree regressor (or bootstrap aggregation) uses random subsets of the data to generate estimates, which are then aggregated to form a final prediction [51]. A good implementation and discussion of bagging regressors is available from the Python scikit-learn module [52].

### 2.4. Geospatial Analysis, State and County Heart Failure Admission Rates

Hospital data were aggregated at the state and county levels and then merged with geospatial data under the presumption that HF admissions may have a geographical explanation. All states had admissions, so no data were missing. Some counties had no admissions likely due either to lack of hospital facilities or small populations. A single principal component was used for the 6 workload variables as before, accounting for 98% of the variability at the state level and 97% of the variability at the county level. 

Geospatial maps for the rates of HF incident rates for the selected DRGs from 2016 through 2018 were generated at the county and state levels. Rate data adjust for population changes, allowing comparison of incidence rates across counties or states. Population data for each county and the states by year came from Census Bureau estimates [45]. 

The determination about whether to use spatial regression/error models (see Mahara et al. [53]) or simple spatial mapping was informed by residual diagnostics of regression models (i.e., Moran’s I global test of residuals with post-hoc Lagrangian multiplier diagnostics as required). In all cases, maps of regression residuals are mapped to provide a visual indicator of outliers [54]. Spatial models are compared to non-spatial models for both coefficients and performance metrics (i.e., R^2^). Spatial contiguity was modeled using Rook (edge borders considered neighbors) and Queen (edge and vertex borders considered neighbors) criteria. Only first-order Rook and Queen criteria were evaluated. Further, we used the row-standardized sums for weighting neighbors and a zero-policy that allowed for weight vectors of zero length for areas with unconnected neighbors. 

### 2.5. Changes in DRGs

The significance of changes for 2016 through 2018 (DRG rates) are also evaluated by a non-parametric Friedman’s test. The Wilcoxon non-parametric test is preferable and more conservative than repeated samples ANOVA, as normality, homogeneity of variance, and independence assumptions do not hold [55]. 

### 2.6. Software 

All analysis was performed in Anaconda Python Release 3.8 [56], R Statistical Software, and Microsoft Excel 2016 [29]. Python was used primarily for tree models, while R provided regression analysis and geospatial analyses. The primary geospatial packages included *tmap* [57], *sf* [58], *sp* [59], and *spatialregression* [59]. Online interactive maps were done in the R package, *leaflet* [60]. 

## 3. Results

### 3.1. Descriptive Statistics—Quantitative Data

Descriptive statistics for all data are freely available online at https://rpubs.com/R-Minator/heart [61]. A roll up for the quantitative data is provided in Table 2. The average hospital observation during any given year had approximately 1600 observations of DRG 291, 292, and 293 (median of 383). That same hospital had approximately 147 staffed beds (median of 86), 7 thousand discharges (median of 2.8 thousand), and approximately 6.4 thousand surgeries (median of 4.5 thousand). The average hospital had positive income (in millions) of $17.3 (median of $2.03), significant cash on hand ($20.3 thousand, median of $1.95 thousand), and positive equity. The typical hospital had just over 1000 employees (median of 436) with 232 affiliated physicians (median of 104) and was reimbursed 45% by Medicare (median of 42%). Only 9% reimbursement was from Medicaid (median of 6%). 

**Figure 2:**
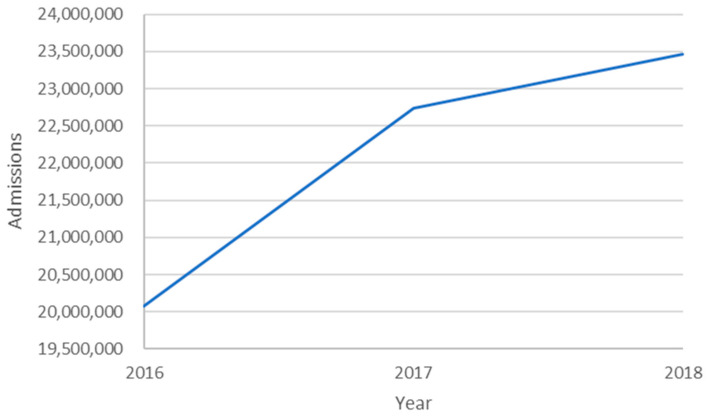
Number of DRGs as a function of year.

**Figure 3:**
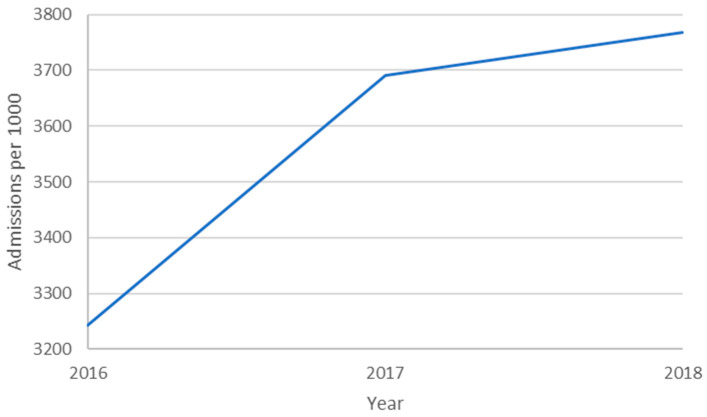
Inpatient diagnoses rates as a function of year.

Year over year, both DRGs and rates of DRGs per 1000 population increased as illustrated in Figure 2 and Figure 3, respectively. The significance of the DRG increase is the financial consideration. The significance of the rate of DRG increase is the epidemiological consideration. If the DRG rate is considered a proxy for incidence rate, then there is either a significant increase, a coding issue, or something else. These considerations are found in the discussion section. One might expect the DRG rate graph to remain horizontal (static). Independent variables remained relatively constant year over year likely due to repeated measures on the same facilities. 

### 3.2. Descriptive Statistics—Categorical Data

California, Texas, and Florida had the largest number of diagnoses for all years and year over year, largely due to population size, with averages of 1.7 million, 1.6 million, and 1.5 million, respectively. When adjusted per 1000 population, the District of Columbia, West Virginia, and Delaware dominated the with total rates per 1000 population of 109, 103, and 94, respectively. Utah, Hawaii, and Colorado had the smallest average rates, 26, 29, and 35, respectively. 

Most hospitals were in urban settings (58%). Fifty-two percent were voluntary non-profits with 29% proprietary and 18.7% governmental. The vast majority (75%) had no affiliation with a medical school and were short-term care facilities (60%). Nearly no hospitals were classified as Department of Defense (DoD) or children’s hospitals. 

### 3.3. Descriptive Statistics—Financial Estimates

In Fiscal Year (FY) 2008, the Centers for Medicare and Medicaid (CMS) estimated that HF DRGs 291, 292, and 293 national average total costs per case were $10.235, $6.882, and $5.038 thousand, respectively. By FY 2012, CMS increased those estimates to $11.437, $7.841, $5.400 thousand, respectively. In four years, the accumulation rates (1 plus the inflation rate) were 1.139, 1.117, and 1.072 for the DRGs in descending order. Using these accumulation rates, estimates for 2016, 2017, and 2018 were generated. Table 3 shows these extrapolated estimates. 

Another method for estimating these costs involved the use of the Federal Reserve Bank of Saint Louis (FRED) producer price index for general medical and surgical hospitals [62]. The annual accumulation rates for 2013 through 2018 were estimated as 1.022, 1.012, 1.007, 1.013, 1.018, and 1.023, respectively. Applying these to the 2012 total costs from CMS results in Table 4 estimates for 2016 through 2018.

**Figure 4:**
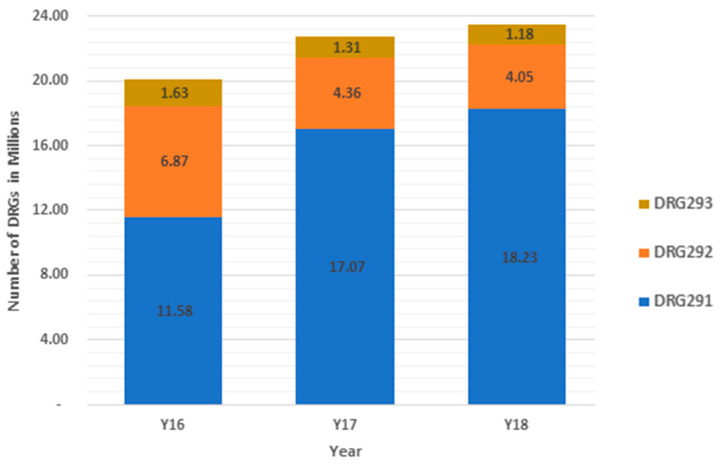
Number of DRGs by type (left axis) and cost estimates by DRG type and total, 2016 through 2018.

**Figure 5:**
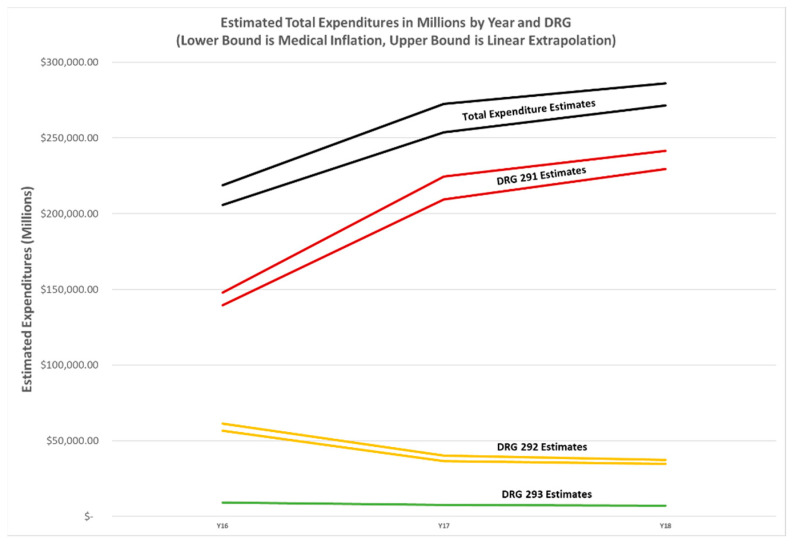
Associated cost estimates in billions (total and by DRG) per year.

Both estimates are reasonably close. To estimate costs, we used both of these tables separately as upper and lower bounds. Since these total costs represent only CMS costs, the actual financial burden across all payers is likely underestimated as commercial third-party insurers can reimburse up to 90% more than Medicare for the same diagnosis [63]. Figure 4 illustrates the number of DRGs by year, while Figure 5 shows the associated aggregate cost estimates. 

In Figure 4, it is clear that DRG 291, the DRG with the highest average reimbursement rate per case, has increased non-linearly, while DRG2 292 has seen a small drop, and DRG 293 is flat. In Figure 5, the total cost estimates for 2018 are nearly $66 billion more than 2016 on average. DRG 291, the most expensive DRG, has seen reimbursement increases of $92 billion on average. Reasons for such an increase are explored in the discussion section. 

### 3.4. Descriptive Statistics—Correlational Analysis

**Figure 6:**
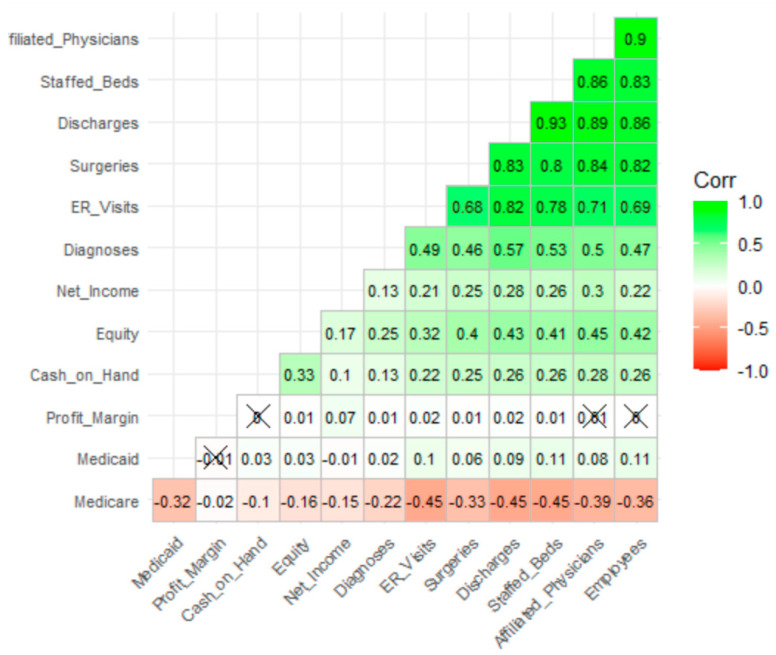
Hierarchical clustered correlation of quantitative variables.

Hierarchical clustered correlation analysis of quantitative variables (Figure 6) illustrates tight relationships among many variables. This type of correlation analysis clusters variables based on distance measures (e.g., Euclidean), so that those which are most highly correlated are close in location. These variables are then placed into a correlation plot or correlogram. Figure 6 illustrates that discharges and staffed beds are most closely associated with the number of diagnoses, our primary variable of interest. More importantly, the workload variables appear to have significant collinearity that must be addressed for regression-based models.

**Figure 7:**
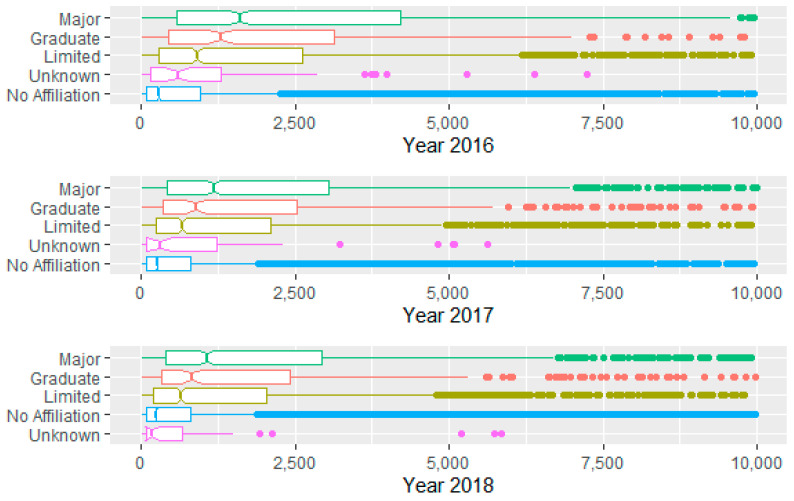
Number of diagnoses by year by medical school affiliation (some outliers omitted).

**Figure 8:**
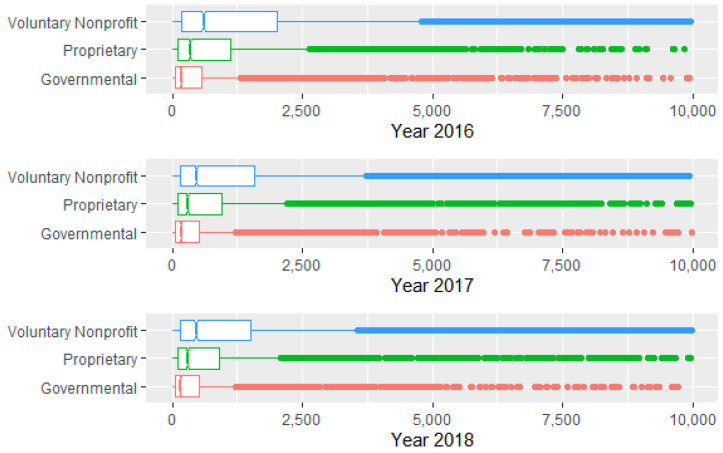
Number of diagnoses by year by type of hospital (some outliers omitted).

Analysis of the relationship between some categorical variables and the number of diagnoses also proved interesting. Notched boxplots by year and medical school affiliation reveal that hospitals with major medical school affiliations experience a larger median number of diagnoses at the 0.05 level, a result that is to be expected. (See Figure 7). Further, voluntary not for profits see a larger median number of diagnoses (Figure 8).

### 3.5. Explanatory Models for Heart Failure Diagnoses, Hospital Unit of Analysis

#### 3.5.1. Regression Models

Linear, lasso, and elastic net regression evaluated the number of diagnoses as a function of all other variables. Models built on the training set and applied to the training and test sets resulted in predicted R^2^ values of 0.501, 0.328, and 0.417 (training) and 0.454, 0.323, 0.348 (test) for the OLS, lasso, and elastic net models, respectively. The OLS model predicted better than the constrained regression models and did not overfit. Appendix B provides the coefficient estimates for all variables after fitting on the entire dataset ($F_{73,\;40183}=534,\;p<0.001,\;\;R^{2}=0.492$). 

Very few coefficients are recommended by the lasso and elastic net models. The lasso model suggests that the workload principal component and DRG 293 are important predictors, both of which are associated with reduced diagnoses ceterus parabus. Elastic net was similar in recommending inclusion of workload as well as DRG 292 and DRG 293, all associated with reduced diagnoses. The OLS model had a larger array of variables that were statistically significant, and the coefficients of the largest magnitude for the min-max scaled variables were associated with workload (−0.439), Short-Term Acute Care hospitals (STAC, 0.084), cash on hand (0.082), and Critical Access Hospitals (CAH, 0.082). When evaluated by categorical groups, the most significant variables were workload (0.302 additional R^2^), DRGs (0.162 additional R^2^), and hospital type (0.011 additional R^2^). 

#### 3.5.2. Tree Ensemble Models

Random forests, extra trees regression, gradient boosting, and bagging regressors after some hyperparameter tuning on the training set predicted HF diagnoses on the test set with reasonable accuracy (R^2^ = 0.829, 0.862, 0.821, and 0.830, respectively). The number of trees used for each estimator was tuned along with the maximum depth of the trees (number of branches). A pseudo-random number ensured that any model improvements were not due to the random number stream. All models accounted for more variance than any regression model. 

**Figure 9:**
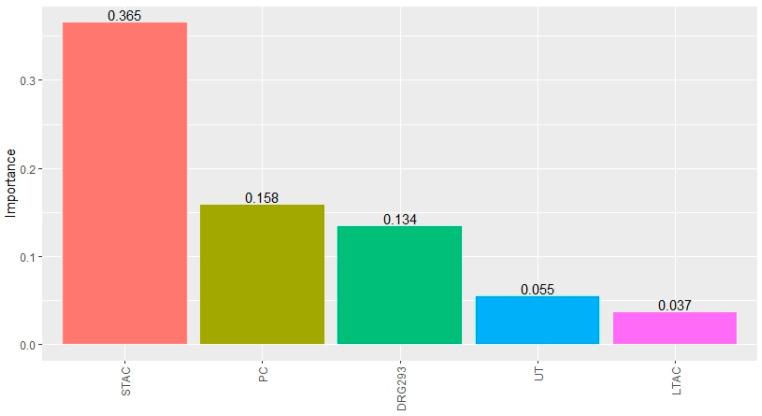
Variable importance measures from the extra trees regression model. STAC = Short-Term Acute Care, PC = principal component for workload, UT = the state of Utah, and LTAC = Long-Term Acute Care.

The best-performing tree ensemble was the extra trees regression. This model ensembled 50 trees and resulted in variable importance shown in Figure 9. Similar to the regression models, hospital type (STAC/LTAC), workload (PC), and DRG (DRG 293) were important along with the state of Utah. 

The conclusion for both the regression and tree models is that hospital-level diagnoses by DRG are forecastable and that workload along with hospital type are important in doing so. Further, the models indicated that geography might be important, as individual state variables and urban/rural status were important to the OLS and the tree models. These models were evaluated on the hospital unit of analysis for raw diagnoses numbers. Rate-based admission models were then evaluated for the states and counties. 

### 3.6. State-Level Geospatial Analysis

A descriptive analysis of HF from 2016 through 2018 using geographical information systems was conducted to evaluate regional differences. Primarily, we were interested in rates per standardized unit in the population of the geographical area. Populations were based on Census Bureau estimates for each geographic region [44,45]. The state-level analysis was limited in that only 50 states and Washington, D.C. were included (*n* = 51). 

**Figure 10:**
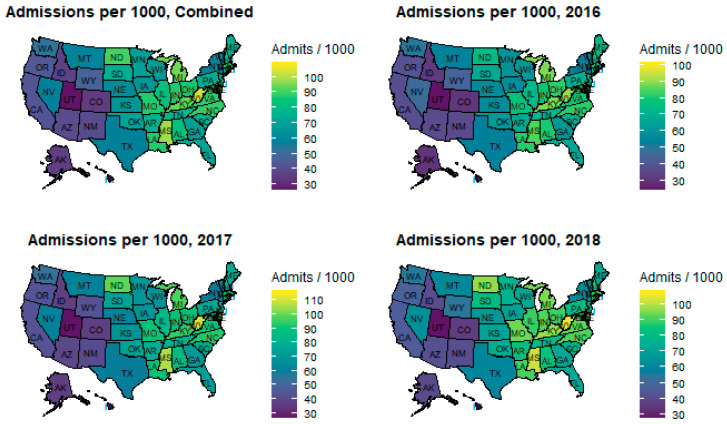
Admissions per 1000, 2016 through 2018.

Results of the state analysis are available here: https://rpubs.com/R-Minator/HeartState [64]). There is a clear bifurcation in the center of the United States separating high and low rates (see Figure 10). That bifurcation suggests a clear West–East difference, favoring the West Coast. Washington, D.C. has experienced the highest average admission rate for diagnoses of HF (109.5 per 1000), which might be due to the large presence of military and veteran care facilities) followed by West Virginia (102.8 per 1000), Mississippi (98.2 per 1000), Michigan (94.3 per 1000), Delaware (94.2 per 1000), Kentucky (93.8 per 1000), North Dakota (90.6 per 1000), North Carolina (88.7 per 1000), Virginia (88.0 per 1000), and Missouri (87.5 per 1000). Of interest is that previous studies indicate that these states also see many admissions due to the opioid crisis [41]. 

**Figure 11:**
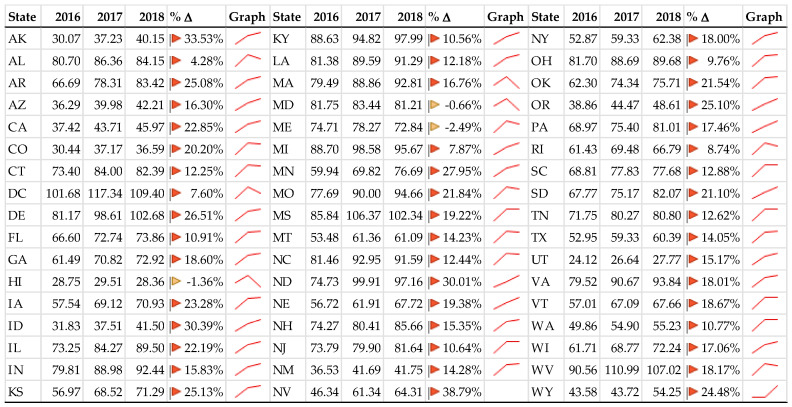
Diagnoses per 1000 by year by state.

From 2016 through 2018, the average rate of diagnoses per 1000 population increased for nearly all states. A Friedman rank sum test (paired, non-parametric ANOVA) of rates by state by year revealed significantly different rates by year by state (χ^2^_2_=70.941, *p* < 0.001). Figure 11 illustrates the changes by year and by state. 

Further, evaluating obesity prevalence intensity from the Centers for Disease Control and Prevention (CDC) shows significant correlation between obesity and DRGs per 1000 [65]. A Spearman’s test for correlation of obesity prevalence and 2018 DRGs per 1000 was statistically significant with ρ = 0.689, S = 6867.7, *p* < 0.001. 

**Figure 12:**
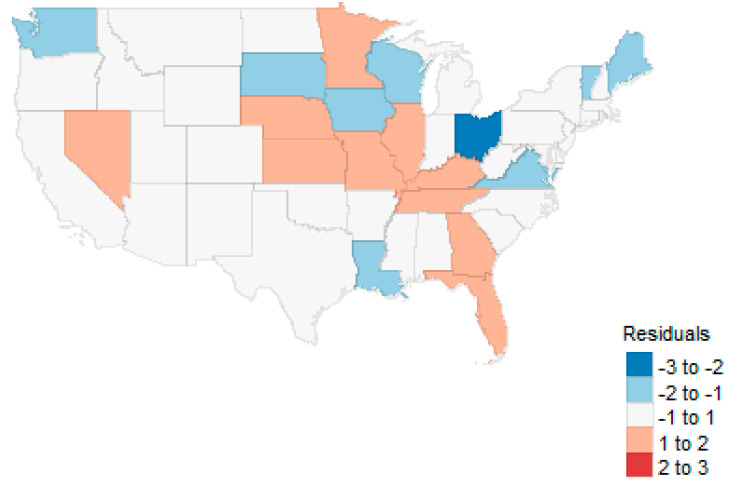
Map of regression residuals.

Ordinary least square regression was performed on the state admission rate as a function of the quantitative, aggregated variables. While the model was statistically significant and accounted for reasonable variability $\left(F_{10,\;40}=4.5,\;p<0.001,\;R^{2}=0.529\right)$, only the proportion Medicare was significant at the 0.05 level. Most important to this preliminary analysis was whether state-level spatial data were important to evaluating admission rates. The spatial map of the standardized residuals [57] as well as residuals associated separate linear models for all included variables is available as an interactive GIS map here: https://rpubs.com/R-Minator/heart [61]. The spatial residuals shows some spatial correlation (see Figure 12). The visual check was confirmed by a global test for both Queen and Rook neighbors suggest that spatial relationships exist, (I = 0.309, *p* < 0.001 and I = 0.306, *p* < 0.001, respectively) [66]. Lagrangian multiplier diagnostics (non-robust and robust) suggested that the preferred models would be spatial lag rather than spatial error, as robust tests for error models were insignificant while lag models remained significant (see [61]).

Generalized spatial two-stage least squares estimated Queen, and Rook models, while a comparison linear model was estimated in traditional fashion. R^2^ for OLS, Queen, and Rook models were 0.529, 0.816, and 0.809, respectively. Queen and Rook models performed better on the state-aggregated data. The coefficient results of the spatial models were nearly identical to each other, while the OLS was obviously needed geospatial data to improve its performance (see Table 5). The geographic component for Queen and Rook models were statistically significant along with mean profit margin and the proportion of facilities with major medical school affiliation were important in predicting diagnoses rates.

### 3.7. County-Level Spatial Analysis

#### 3.7.1. Maps

The average three-year HF admissions per 1000 county population are shown in the interactive map online [67]. These county maps show that the admissions are generally (as expected) in large metropolitan areas, e.g., Dekalb, Illinois (0.65 per 1000). There are exceptions, however. For example, Montour, Pennsylvania is a small county that is home to a large Geisinger facility and thus has a higher than expected admission rate (1.00 per 1000).

**Figure 13:**
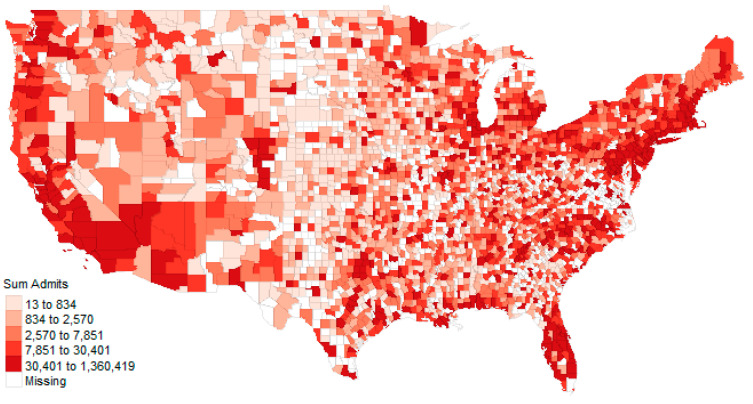
The total admissions from 2016 through 2018 by county.

**Figure 14:**
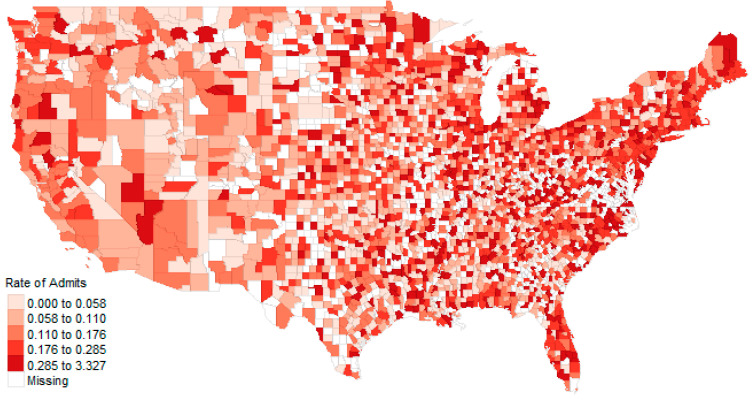
The admission rate from 2016 through 2018 by county.

The top ten counties for average rates per 1000 over three-years were Winchester, Virginia (3.33 per 1000); Norton, Virginia (3.21 per 1000), Montour, Pennsylvania (3.01 per 1000); Fredericksburg, Virginia (2.13 per 1000); DeKalb, Illinois (1.95 per 1000); Harrisonburg, Virginia (1.58 per 1000); Petersburg, Virginia (1.57 per 1000); Boyd, Kentucky (1.45 per 1000); St. Francois, Missouri (1.34 per 1000); and Adams, North Dakota (1.21 per 1000). Of interest is that half of these counties are in the state of Virginia, perhaps due to the large military and veteran medical centers located in the area. Many of these counties (e.g., Winchester) are small but have large healthcare facilities. Figure 13 and Figure 14 illustrate the raw admissions and rate of admission per 100,000 population, respectively. Other maps are available online.

#### 3.7.2. Regression Models, County-Level of Analysis

Similar to what was done at the state level, an exploratory spatial regression model using first-order Queen and Rook contiguity criteria to evaluate the importance of geography was performed using rolled, Z-scaled, county-level independent variables on the county-level admission rate variable (admissions per population in each county). Moran’s I global test suggested that the OLS model was probably sufficient (I = 0.02, *p* = 0.100); however, we explored further with Lagrangian multiplier diagnostics. The robust from of these statistics slightly favored a lag model. Results of the regression are in Table 6, and the residual maps for the global model and the individual variables are available online [61] The OLS, Queen, and Rook regression models accounted for only a small fraction of the sum of the squares (*R^2^* = 0.169, *R^2^* = 0.132, *R^2^* = 0.132, respectively). 

Most variables in all models are statistically significant largely due to the sample size, but the coefficients are of small magnitude. Every variable except for the proportion Medicaid was statistically significant in the best-fitting OLS model, and yet the magnitude of the coefficients across the three models (OLS, Queen, Rook) was quite similar. Profit margin, and cash on hand were negatively associated with admission rates in the OLS model, ceterus parabus. All other variables had positive coefficients in the OLS model. Interpretation of directionality must be done cautiously, as the variables act together in prediction. 

Interactive maps of the admission rate, model residuals (OLS, Queen, and Rook), as well as residuals for individual variables are provided online [61]. The residual maps are not suggestive of spatial autocorrelation given the residual dispersion by county. Future explanatory models can omit spatial correlation.

Given the small contribution of the OLS, Queen and Rook models to estimating county-level admission rates, ensemble models were investigated at the county level. With 2431 valid observations, sufficient power existed to split the data into training and test sets (80%/20%). Results of hyperparameter tuned models suggested that extreme gradient boosting was the best model as the predictive R^2^ for random forests, extra trees, extreme gradient boosting, and bagging regressors was 0.242, 0.292, 0.264, and 0.130, respectively. The first three models performed much better on the 20% withhold set than regression models. The variable importance analysis suggested that workload, cash on hand, and mean equity were the most important variables with importance scores of 0.35, 0.13, and 0.13, respectively.

## 4. Discussion

### 4.1. Review of Findings

From Figure 2, we can see that the number of DRGs for HF is increasing over time. We do not have sufficient data or monthly data to run time series analyses such as exponential trend seasonality and autoregressive integrated moving average models. Even without those models, it is clear that there appears to be an increase in HF admission diagnoses and a change in intensity from 2016. What is most interesting is that intensity changes are largely in the North Central while current incidence rates are highest East of the Texas panhandle.

The heterogeneity found in this study and others inside the United States [68] is present internationally. One study found high variation in spending based on economic status of the country [15]; other studies have shown heterogeneity in prevalence rates (with a predominant global range of 1–3%) [69]. The reasons for heterogeneity are varied; however, income disparity appears to be a major contributor [70].

Our results indicate there has been a significant shift in cardiology diagnoses since 2016. As we note, it is clear that DRG 291, Heart Failure and Shock with Major Complication of Comorbidity (MCC), counts and costs have increased non-linearly. DRG 292, Heart Failure and Shock with Complication or Comorbidity (CC), has seen a small drop and DRG 293, Heart Failure and Shock without Complication or Comorbidity (CC)/Major Complication or Comorbidity, is flat. A DRG is determined by the principal diagnosis, the principal procedure, if any, and certain secondary diagnoses identified by CMS as comorbidities and complications (CCs) and major comorbidities and complications (MCCs) [71]. A comorbidity is a condition that existed before admission. A complication is any condition occurring after admission, not necessarily a complication of care [72]. Although HF DRGs represented the largest cause of hospitalizations among Medicare beneficiaries and were among the costliest to Medicare prior to 2016, the results of our study now suggest that total cost estimates for these three DRGs in 2018 are now nearly $61 billion more than 2016 [73,74,75]. DRG 291, the most expensive DRG, is associated with $91 billion cost increases from 2016. 

Tree models at the hospital unit of analysis were capable of capturing close to 90% of the variability on a 20% withhold test set. The variables of most importance to this prediction were consistent with the variables found through regression modeling. Specifically, hospital type and workload variables captured by a single principal component re important in predicting the number of diagnoses by facility. This finding is particularly useful in that localities and states may forecast expected costs associated with the ever-increasing number of HF admissions with hospital-level granularity.

For the state-level GIS analysis, OLS as well as first-order Queen and Rook models were estimated, and the coefficients were both stable and congruent across models. Facility ownership and hospital type were important predictors, and the first-order Queen model provided the best variance capture (R^2^ = 0.816). No sophisticated predictive models were available due to the small samples size (50 states plus the District of Columbia). 

At the county level, no regression models performed well, although the OLS model captured more variance than the first-order Queen and Rook models. In fact, the best-performing model after hyperparameter tuning was an extreme gradient-boosted tree ensemble that captured 36% of the variability on an unseen test set.

### 4.2. Limitations and Future Work

This study is limited in that only three complete years of data were available. As more data become available, the analysis will be expanded. Further, the study does not consider sub-DRGs, which might provide additional value in understanding the cost structure, particularly since procedures such as extracorporeal membrane oxygenation (ECMO) are highly costly yet coded across multiple DRGs.

While it is likely that many individuals receiving care in a geographic area are from outside that county or state, the majority are likely to receive care near the vicinity of the admission, particularly since HF may result in a medical emergency. Further, the intent of the study is to explain admissions and their associated treatment locations. For public health professionals interested in where HF events (rather than admissions) occur, the state-level geospatial analysis would be more reflective as it reduces bias associated with facility locations.

Although our research has demonstrated substantial reliability in the explanatory factors associated with the longitudinal growth trajectory, it does not explain the reasons why we see such substantial growth in DRG 291 versus DRGs 292 and 293. Given our study results, there are several potential drivers that could meaningfully contribute to the growth in DRG 291 from 2016 through 2018. First, there may have been a significant increase in patients with cardiac conditions and additional major comorbidities. This cannot be simply dismissed given the rapid increase in Medicare eligible beneficiaries—by some estimates as many as ten thousand per day—and the prevalence of obesity, chronic obstructive pulmonary disease, and other age and lifestyle-related conditions [76,77,78]. However, given the relatively flat or declining rate in DRGs 292 and 293, we do not believe this is the only driver of our findings. Our findings support other predictions that soon patient demand will outpace the supply [79,80].

Second, up until October 2018, all extracorporeal membrane oxygenation (ECMO) cases were assigned to DRG 003, which typically reimburses at a rate of approximately $100,000 per case [81]. In fiscal year 2019 (beginning October 2018), that reimbursement methodology changed so that every ECMO case would no longer be assigned to DRG 003. Rather, the DRG assigned depends on the path of the cannulation. If the ECMO patient is accessed centrally, DRG 003 is still applied. However, if cannulated peripherally, then it falls into another (lower-paying) DRG [82,83]. Although there is only a three-month overlap of this change and our study dataset, there is high likelihood this additional volume is reflected in our study in 2018. 

Third, since 2010 and the passage of the Affordable Care Act, many cardiologists have sought hospital employment versus private practice. The uncertainty of continued healthcare reform efforts, burdensome electronic health record costs, declining CMS reimbursement rates in physician professional fees for non-invasive testing procedures (e.g., electrocardiograms and nuclear stress tests), and younger clinicians’ different expectations related to work and personal life balance have all combined to prompt cardiology groups to seek ways to stay financially viable. Today more than 70 percent of cardiologists are employed by hospitals or health systems [84,85]. Hospitals, in turn, seek to maximize utilization and reimbursement from the highly resource intensive cardiology service lines. Prior research from the National Bureau of Economic Research found that hospitals responded to price changes by up-coding patients to diagnosis codes associated with large reimbursement increases. These authors indicate hospitals do not alter their treatment or admissions policies based on diagnosis-specific prices; however, they employ sophisticated coding strategies in order to maximize total reimbursement [86,87].

Fourth, we suspect the recent transition from ICD-9 to ICD-10 that occurred in October 2015 is a contributing factor. Starting on 1 October 2015, there were 68,069 valid ICD-10-CM diagnosis codes, representing a nearly 5-fold increase from the 14,025 valid ICD-9-CM diagnosis codes. ICD-10-CM diagnosis codes are structured differently from ICD-9-CM codes and provide more detail [49]. This code expansion allows providers the ability to capture the severity and specificity of the condition in much greater detail, which may prompt increased use of DRG 291. 

As we look at the number of times many of the codes are being assigned to any particular patient, we see a notable change in how physicians are diagnosing. Previously, we had an ICD-9 diagnosis code with some generic areas that covered many patients. A very general and generic set of HF codes existed under 428.x in ICD-9. There was little specificity as to sidedness of the issue or specifics of the disease. ICD-10 codes allow a specific diagnosis per code, and these codes will continue to change over time due to physicians’ adaptation of coding in this manner. For example, the I50.8xx codes did not exist in 2016, but they have been used since 2017, with another change adding more sub-codes in 2018.

Today, we have specific codes for specific diseases and processes which go on within the heart, to include acute on chronic concerns as well. The adjustment to ICD-10 codes has undoubtedly created a learning phase for practitioners on determining the appropriate codes as well as when and how to use them. 

We would expect to see some elevation from year to year with the growth of the Baby Boomer population coming into healthcare, without an age adjustment to the population. This is shown in the numbers from 2016 through 2018 with total admissions diagnoses increasing from 5.39 M to 5.61 M to 5.69 M. However, how the diagnosis codes are being applied shows variation from year to year, to include some years of negative numbers in several codes. Many of the negative values for codes are for “unspecified” types of heart disease. This shows that we are moving away from generic diagnoses and towards diagnoses based in specificity instead, which is one of the purposes of moving to ICD-10.

One could draw a conclusion of upcoding: a monetary free for all, assigning diagnoses based on what pays the most. However, in many cases the physician is not billing based on a diagnosis code, but on the level of the visit and the type. This is obviously dependent upon insurance types, contracts, and other inputs outside the discussion level of this paper.

## 5. Conclusions

The policy implications of this analysis are several. First, clearly there is a need to continue to focus on a population health approach to reduce obesity rates across the country, specifically focusing on the geographic states identified with the highest incidence and prevalence across the study timeline. The substantial increase in DRGs 291–293 show that shifting funding to prevention from chronic disease management certainly has the financial evidence to support this approach. The argument is certainly made that education is not sufficient to change lifestyle and behaviors contributing to the rise of heart disease shown here, so it is time to begin exploring a punitive annual health assessment requirement for high-risk individuals who fail to make significant risk factor changes. While a punitive health assessment might incentivize behavioral modifications and might result in lower costs, there is also the possibility that these modifications will possibly require medication and surgical interventions. Using such a strategy alone is not likely to produce the results required. The health administrator will certainly need to analyze both the volume and scope of services within these analyzed DRGs to ensure the evident increase in demand indicated will be available, specifically in the identified high incidence geographic areas. In Certificate of Need (CON) states, this analysis will be beneficial in getting the CON approved based on the increased demand. Evidence shows that CON states for cardiac services, of which most of the high incidence and prevalence states in the study are, have higher mortality rates for cardiac services [88]. Another significant potential policy implication is a continued re-evaluation of the need for CONs in general. Researchers are now questioning whether they are needed, as they restrict services and lead to increased mortality [89]. A final policy implication relates to policy incentives. Since heterogeneities in HF prevalence and HF facilities exist (i.e., center of the country versus the North and East), provider incentive policies might be established at both the federal and local levels to balance healthcare professional supply with patient demand.

## Figures and Tables

**Table 1:** Independent variables.

**Technical Variables**	**Defined**	**Measurement**
% Medicare	Percent of patients reimbursing via Medicare	Ratio
% Medicaid	Percent of patients reimbursing via Medicaid	Ratio
Diagnostic-Related Groups	DRG 291, DRG 292, DRG 293	Categorical
Ownership	Hospital Ownership	Categorical
Medical School Affiliation	None, Limited, Major, Graduate Affiliation	Categorical
Hospital Type	Children, Critical Access, Long-Term, Psychiatric, Rehab, Short-Term	Categorical
**Workload Variables**	**Defined**	**Measurement**
Discharges	Number of patients discharged from admission	Integer
ER Visits	Number of emergency room visits	Integer
Affiliated Physicians	Number of physicians affiliated with hospital	Integer
Employees	Number of direct employees of hospital	Integer
Staffed Beds	Number of staffed beds operated by hospital	Integer
Surgeries	Number of surgeries performed	Integer
**Financial Variables**	**Defined**	**Measurement**
Net Income	Net revenues minus loss	Ratio
Operating Profit Margin	Profit divided by revenue	Ratio
Cash on Hand	Cash available to the organization	Ratio
Equity	Assets minus liabilities	Ratio
**Geospatial Variables (and Time Window)**	**Defined**	**Measurement**
State	Indicator variables for hospital’s state	Dichotomous
County	Indicator variables for county in states	Dichotomous
Urban/Rural	Indicator variable for metropolitan status	Dichotomous
Year	Indicator variables for year of observation (2016 through 2018)	Dichotomous

**Table 2:** Descriptive statistics for the study (dollars in millions).

*n* = 40,257	Mean	SD	Median	Min	Max
Number DRGs	1640.258	3334.942	385	11	57,461
Staffed Beds	146.507	172.468	86	2	2753
Affiliated Physicians	231.786	353.461	104	1	4328
Employees	1008.034	1683.991	436	4	26,491
Percent Medicare	0.448	0.186	0.422	0	0.983
Percent Medicaid	0.087	0.091	0.063	0	0.869
Discharges	7014.259	9908.036	2811	1	129,339
ER Visits	32,864.497	33,976.188	25,085	0	543,457
Surgeries	6349.317	7987.273	4464	0	130,741
Net Income ($ in M)	$17.23	$117.65	$2.04	−$1.21	$33.01
Cash on Hand ($ in M)	$20.28	$120.24	$1.99	−$2.51	$3.88
Profit Margin	−0.03	1.25	−0.02	−15.45	62.07
Equity ($ in M)	$174.11	$625.76	$33.94	−$3.25	$10.24

**Table 3:** Estimated total costs for heart failure by DRG in thousands, linear extrapolation method.

DRG	2016	2017	2018
DRG 291	$12,780	$13,155	$13,243
DRG 292	$8934	$9245	$9257
DRG 293	$5788	$5891	$5998

**Table 4:** Estimated total costs for heart failure by DRG in thousands, medical inflation rate method.

DRG	2016	2017	2018
DRG 291	$12,058	$12,273	$12,582
DRG 292	$8267	$8414	$8626
DRG 293	$5693	$5795	$5491

**Table 5:** Results of the state-level regression.

Variable	Linear Model		Queen Model		Rook Model	
Rho			0.993	***	0.993	***
(Intercept)	−0.221		−0.101		−0.123	
Income	−0.055		0.011		0.01	
Profit Margin	−0.418		−0.458	**	−0.458	**
Cash on Hand	−0.162		0.015		0.023	
Equity	0.183		0.060		0.049	
% Medicare	0.842	***	0.221		0.24	
% Medicaid	−0.163		0.058		0.061	
% Non-Profit	0.129		−0.128		−0.122	
% Med School	0.386		0.398	***	0.408	***
% STAC	0.483	**	−0.016		−0.015	
Workload	−0.004		−0.162		−0.152	

** *p* < 0.01 and *** *p* < 0.001.

**Table 6:** Regression table for county analysis.

Variable	Linear Model		Queen Model		Rook Model	
Rho			−0.539	***	−0.538	***
(Intercept)	0.019		0.048	***	0.047	***
Income	0.010		0.015		0.015	
Profit Margin	−0.007	*	−0.002		−0.002	
Cash on Hand	−0.063	*	−0.058	***	−0.057	***
Equity	0.090	*	0.081	***	0.081	***
% Medicare	0.049	**	0.050	***	0.050	***
% Medicaid	0.012	*	−0.001		−0.001	
% Non-Profit	0.013	**	0.016	***	0.016	***
Mean Affiliated Providers	0.045	**	0.044	***	0.044	***
% STAC	0.041	**	0.044	***	0.044	***
Workload	0.084	*	0.079	***	0.079	***

Moran’s I favors the linear model, but all coefficients are similar; * *p* < 0.05, ** *p* < 0.01, and *** *p* < 0.001.

## Data Availability

The dataset(s) supporting the conclusions of this article are available in the Definitive Healthcare repository, https://www.definitivehc.com/ [43]. All Python and R code are available here: https://rpubs.com/R-Minator/heart [61], https://rpubs.com/R-Minator/HeartState [64], and https://rpubs.com/R-Minator/HeartCounty [67].

## Appendix A

List of Abbreviations	
ER	Emergency Room
CC	Complication of Comorbidity
CMS	Centers for Medicare and Medicaid
CON	Certificate of Need
DRG	Diagnostic-Related Group
ECMO	Extracorporeal Membrane Oxygenation
GIS	Geographical Information System
HF	Heart Failure
HFpEF	Heart Failure with preserved Ejection Fraction
HFrEF	Heart Failure with reduced Ejection Fraction
ICD	International Classification of Disease Version (-version)
MCC	Major Complication of Comorbidity

## Appendix B

**Table A1:** Results of regression analyses for the number of diagnoses, hospital unit of analysis.

Variable	Linear		Lasso	Elastic Net	Variable	Linear		Lasso	Elastic Net
Workload	−0.439	***	−0.298	−0.323	State_MA	0.010	**	0.000	0.000
Net Income	−0.043	***	0.000	0.000	State_MD	0.013	***	0.000	0.000
Profit Margin	0.026	***	0.000	0.000	State_ME	−0.002		0.000	0.000
Cash on Hand	−0.082	*	0.000	0.000	State_MI	0.013	***	0.000	0.000
Equity	0.012	***	0.000	0.000	State_MN	0.003		0.000	0.000
% Medicare	0.029	**	0.000	0.000	State_MO	0.005		0.000	0.000
% Medicaid	−0.002	***	0.000	0.000	State_MS	0.003		0.000	0.000
Proprietary Ownership	0.003		0.000	0.000	State_MT	0.004		0.000	0.000
Non-profit Ownership	0.006	***	0.000	0.000	State_NC	0.016	***	0.000	0.000
Limited Med Sch Aff	0.002	***	0.000	0.000	State_ND	0.004		0.000	0.000
Major Med Sch Aff	−0.009		0.000	0.000	State_NE	0.001		0.000	0.000
No Med Sch Aff	0.000	***	0.000	0.000	State_NH	0.002		0.000	0.000
Unknown Med Sch Aff	−0.005		0.000	0.000	State_NJ	0.012	***	0.000	0.000
Critical Access Hospital	0.074		0.000	0.000	State_NM	−0.002		0.000	0.000
DoD Hospital	0.000	***	0.000	0.000	State_NV	0.007		0.000	0.000
LTAC Hospital	0.048	***	0.000	0.000	State_NY	−0.005		0.000	0.000
Psych Hospital	0.067	***	0.000	0.000	State_OH	0.007	*	0.000	0.000
Rehab Hospital	0.058	***	0.000	0.000	State_OK	0.001		0.000	0.000
STAC Hospital	0.084	***	0.000	0.006	State_OR	−0.002		0.000	0.000
State_AL	0.005		0.000	0.000	State_PA	0.002		0.000	0.000
State_AR	0.003		0.000	0.000	State_RI	0.002		0.000	0.000
State_AZ	−0.002		0.000	0.000	State_SC	0.006		0.000	0.000
State_CA	0.000		0.000	0.000	State_SD	−0.002		0.000	0.000
State_CO	−0.003		0.000	0.000	State_TN	0.003		0.000	0.000
State_CT	0.013	**	0.000	0.000	State_TX	0.004		0.000	0.000
State_DC	0.006		0.000	0.000	State_UT	−0.004		0.000	0.000
State_DE	0.018	**	0.000	0.000	State_VA	0.014	***	0.000	0.000
State_FL	0.006		0.000	0.000	State_VT	−0.001		0.000	0.000
State_GA	0.009	**	0.000	0.000	State_WA	0.005		0.000	0.000
State_HI	−0.003		0.000	0.000	State_WI	0.003		0.000	0.000
State_IA	0.001		0.000	0.000	State_WV	0.002		0.000	0.000
State_ID	0.000		0.000	0.000	State_WY	0.002		0.000	0.000
State_IL	0.009	**	0.000	0.000	Urban	0.004	***	0.000	0.000
State_IN	0.006		0.000	0.000	Year 2017	0.003	***	0.000	0.000
State_KS	0.002		0.000	0.000	Year 2018	0.004	***	0.000	0.000
State_KY	0.005		0.000	0.000	DRG 292	−0.040	***	0.000	−0.016
State_LA	0.006		0.000	0.000	DRG 293	−0.056	***	−0.013	−0.029

* *p* < 0.05, ** *p* < 0.01, and *** *p* < 0.001.
